# “From Nares to Wound: Exploring the mechanisms for Staphylococcal surgical site infections, implications for infection prevention”

**DOI:** 10.1017/ash.2023.197

**Published:** 2023-07-26

**Authors:** Darren P. R. Troeman, Jan A. J. W. Kluytmans

**Affiliations:** 1 Julius Center for Health Sciences and Primary Care, University Medical Center Utrecht, Utrecht University, Utrecht, The Netherlands; 2 Department of Medical Microbiology, University Medical Center Utrecht, Utrecht University, Utrecht, The Netherlands

## Abstract

Surgical site infections (SSIs) are important healthcare-associated infections, leading to increased morbidity and mortality, healthcare costs, and prolonged hospital stays. *Staphylococcus aureus* is an important and common microbial cause of SSI. Nasal carriage of *S. aureus* has been shown to be an important determinant for the development of SSI, and interventions aimed at eradicating preoperative nasal carriage are associated with a reduced risk of infection. Yet, it is not entirely clear how the nasally residing *S. aureus* causes SSI at distant body sites. In this commentary, we describe our view on how *S. aureus* can be transported from the nares to the incision site during surgery. In addition, we shed light on the implications of our view for infection prevention research.

Surgical site infections (SSIs) are important healthcare-associated infections, leading to increased morbidity and mortality, healthcare costs, and prolonged hospital stays.^1–4^ SSIs are caused by a variety of microorganisms, depending on the type of surgery that is performed.^5^ In clean surgical procedures, *Staphylococcus aureus* is a common microbial cause of SSI.^6^ *S. aureus* is a commensal microorganism that is present on the skin and mucosal surfaces of humans and different types of animals. Population-based cross-sectional studies have shown that approximately 20–30% of the human population is colonized with *S. aureus*, and longitudinal studies have demonstrated that at least three *S. aureus* carriage patterns can be distinguished in a healthy population: persistent carriers, intermittent carriers, and noncarriers. The primary body site associated with *S. aureus* carriage is the nares of the nose, although *S. aureus* also colonizes other body sites, including the throat, axilla, and perineal area.

A substantial body of evidence has shown that there is a significant association between *S. aureus* colonization of the patient, particularly nasal *S. aureus* colonization, and the occurrence of *S. aureus* infections, including *S. aureus* SSI.^7,8^ In addition, studies have shown that eradication of *S. aureus* colonization by using decolonization treatments, including antiseptic body washes and nasal mupirocin, leads to a substantial decrease in the incidence of *S. aureus* SSIs and other staphylococcal infections.^6,9^ Furthermore, studies that employed genetic characterization of staphylococcal samples, derived from *S. aureus* colonized (including the nose) and infected body sites from the same individuals, have shown that a large proportion of infecting *S. aureus* shares strain type with colonizing *S. aureus*, suggesting an endogenous origin.^10^ For instance, the ASPIRE-SSI study (ClinicalTrials.gov Identifier: NCT02935244), which was a prospective cohort study conducted at 33 sites in 10 European countries and that assessed the incidence and etiological factors for *S. aureus* SSI and bloodstream infections, found that 83% of *S. aureus* SSI in *S. aureus* carriers had probably an endogenous origin (Ref: DPR Troeman MD, D Hazard MSc, L Timbermont PhD et al, 5-2023, submitted for publication). Based on these findings, the question arises of how colonizing *S. aureus* lead to infections at other body sites. Answering this question is crucial to further guide interventions aimed at reducing the incidence and burden of *S. aureus* SSI.

As stated earlier, the most common *S. aureus* carriage site is the nares of the nose. In theory, there are several mechanisms through which *S. aureus* can spread from the nose to the site of infection. With regard to SSI, the bacterial transmission from the nares to the surgical site can occur via one of four routes (or a combination of these routes): (1) direct contact through contaminated skin or mucosal surfaces; (2) indirect contact involving contaminated operating room (OR) equipment or instrumentation; (3) hematogenous transmission from the colonized body site to the surgical site; and (4) airborne transmission via the air within the OR.

## Direct Contact

This occurs when the skin covering the surgical site is colonized by *S. aureus*, and the surgical wound becomes infected when or shortly after the incision is made. It has been shown that the skin of nasal carriers is frequently colonized with *S. aureus* originating from the reservoir in the nose.^8,11^ So, it is safe to assume that nasal carriage leads to skin carriage and that this can subsequently cause infection at distant body sites. However, it is standard practice to use antiseptic agents at the surgical site prior to surgery, which should prevent the development of infection via this route.^12,13^

## Indirect Contact

This can occur when for instance medical equipment or OR personnel that are contaminated with *S. aureus* originating from the nose of the patient, come into contact with the surgical site. However, considering the high level of infection control and especially aseptic conditions during surgery, this transmission route is considered unlikely as a cause of *S. aureus* SSI.

## Hematogenous Transmission

This can occur when *S. aureus* enters the bloodstream while the patient is undergoing surgery. For instance, *S. aureus* may enter the blood circulation following injury of the oropharyngeal mucosal surface during mechanical intubation. Incidental *S. aureus* bacteremia may cause secondary infection of the surgical site. While this is theoretically possible, it is common practice to treat patients perioperatively with antibiotic prophylaxis consisting of broad-spectrum antibiotics with good activity against *S. aureus*.^14,15^ Therefore, this is probably also not a major transmission route in regard to *S. aureus* SSI.

## Airborne Transmission

This can occur when pathogens are carried to the surgical site by small airborne particles, such as droplet nuclei. These pathogen-loaded particles arise during procedures related to the surgery, for instance during inhalation anesthesia, are disseminated by air currents in the OR, and can remain airborne for extended periods of time. Earlier studies have shown that improper OR ventilation can lead to displacement of airborne particles carrying bacteria to the surgical site.^16,17^ In addition, compared to *S. aureus* carriers with lower bacterial loads of colonizing *S. aureus*, patients who are more heavily colonized with *S. aureus* are known to spread more *S. aureus* to their surroundings.^16^ Therefore, airborne transmission could be an important source of infection in *S. aureus* SSI.

Considering the abovementioned arguments, we argue that airborne transmission is currently the most likely route of transmission of *S. aureus* from the nares to the surgical wound, even though we cannot exclude the other options. There is no question that the filtered air that is introduced into the OR is clean and does not contain *S. aureus*. However, there are many factors in the OR that can disturb the airflow during surgery, including OR lights, frequent door openings, patient warming devices, and people. These disturbances cause air turbulence, and this can subsequently lead to contaminated particles being transported to the incision site.^18^ As an example, this online video shows how particles, that are produced near the head of a patient, are transported to the incision site (ref: https://youtu.be/yq-hVBjgZEk). There are also experimental data showing that direct ventilation of the operative site with a mobile laminar airflow unit, which is not influenced by lights etc., does significantly decrease the bacterial contamination of the surgical site.^19^

However, the literature is still ambiguous with regard to the role of airborne transmission in the etiology of SSI. The studies conducted to date assessing the relationship between different air flow types in the OR and SSIs have yielded contradicting results. In addition, a recent meta-analysis found no evidence that laminar flow (which is considered the standard of care in modern surgery) is associated with a lower occurrence of SSI compared to conventional turbulent airflow,^20^ which is consistent with results from earlier reviews.^21,22^ Whilst this is true, it is important to note that the majority of these studies were conducted with national surveillance and registry data, which often lack comprehensive confounder data. In addition, these studies were not conducted with *S. aureus* carriers specifically, which may have diluted the effect.

Based on the above, we conclude that the current evidence does not completely rule out airborne transmission as a major player in the etiology of *S. aureus* SSI in *S. aureus* carriers. Current guidelines, including the WHO guidelines, recommend the use of perioperative decolonization in combination with skin disinfection prior to surgery in nasal carriers of *S. aureus* undergoing high-risk surgical procedures.^23^ While this approach is proven effective and targets the reservoir of the infection, it does not take into consideration the route of transmission. As a matter of fact, current guidelines do not account for airborne transmission at all as a possible transmission route in the etiology of SSI. Therefore, we believe that more research is needed to elucidate the role of airborne transmission in the development of *S. aureus* SSI. For instance, modeling studies assessing the airflow dynamics in the OR under different conditions, or trials including *S. aureus* carriers specifically and assessing the association between different air flow modalities in the OR and the occurrence of *S. aureus* SSI, could inform us about the importance of airborne transmission in the etiology of *S. aureus* SSI. With this knowledge, we will hopefully be better suited to develop more effective antistaphylococcal preventive interventions.
